# Diversity of COVID-19 News Media Coverage across 17 Countries: The Influence of Cultural Values, Government Stringency and Pandemic Severity

**DOI:** 10.3390/ijerph182211768

**Published:** 2021-11-09

**Authors:** Reuben Ng, Yi Wen Tan

**Affiliations:** 1 Lee Kuan Yew School of Public Policy, National University of Singapore, Singapore 119077, Singapore; 2 Lloyd’s Register Foundation Institute for the Public Understanding of Risk, National University of Singapore, Singapore 119077, Singapore; yiwentan@nus.edu.sg

**Keywords:** COVID-19, pandemic, newspapers, cultural values, text as data, public health, public policy, digital humanities, quantitative social science

## Abstract

The current media studies of COVID-19 devote asymmetrical attention to social media; in contrast, newspapers have received comparatively less attention. Newspapers are an integral source of current information that are syndicated and amplified by social media to a wide global audience. This is one of the first known studies to operationalize news media diversity and examine its association with cultural values during the pandemic. We tracked the global diversity of COVID-19 coverage in a news media database of 12 billion words, collated from 28 million articles over 7000 news websites, across 8 months. Media diversity was measured weekly by the number of unique descriptors of 10 target terms of the pandemic (e.g., COVID-19, coronavirus) and normalized by the corpus size for the respective countries per week. Government Stringency was taken from the Oxford COVID-19 Government Response Tracker and cultural scores were taken from Hofstede’s Cultural Values global database. Results showed that Media Diversity Rate increased 6.7 times over 8 months, from the baseline period (October–December 2019) to during the pandemic (January–May 2020). Mixed effects modelling revealed that higher COVID-19 prevalence rates and governmental stringency predicted this increase. Interestingly, collectivist cultures are linked to more diverse media coverage during COVID-19. It is possible that news outlets in collectivist societies are motivated to present a diverse array of topics given the impact of COVID-19 on every segment of society. Of broader significance, we provided a framework to design targeted public health communications that are culturally nuanced.

## 1. Introduction

The current COVID-19 pandemic has impacted multiple facets of society. News media outlets responded by producing a plethora of topics related to the pandemic. Studies of mass media have revealed a rich diversity of news frames of COVID-19 [1,2], but there is a dearth of investigations that explore underlying mechanisms responsible for the diversity in the way COVID-19 is presented in these forms of media. As such, we fill this gap and expand the knowledge of media communication theories and practices by introducing a cultural perspective.

To our knowledge, this is one of the first studies to elicit the association between cultural values and media diversity related to COVID-19. Against this background, this exploration contributes to public health policies by informing policy makers about the intersection between culture and mass media.

In our study, media diversity is defined by how the media provide a wide range of opinions, perspectives, and topics about the pandemic [3]. This entails empowering the public with a range of decisions; subsequently, this exerts a strong influence on public perceptions and behaviors [3]. This is due to the function of mass media, which dampen or amplify societal responses to governmental actions through a diversity of opinions in the media [4]. Agenda setting exemplifies this, where news media sets the tone for dominant narratives in the public’s mind via wide coverage of an issue [5]. Based on these concepts, our study has implications for public policies, informing policy makers about the nature of the relationship between media diversity, cultural values, and the pandemic. This means that policy makers should account for national values when informing the media about how it presents the pandemic to achieve certain aims; for instance, policies can guide media outlets to prime certain cultural values by adjusting the coverage for the pandemic to achieve a particular goal [6,7,8]. This could be used to encourage society to develop resilient and adaptive behaviors to cope with the pandemic [9].

Culture is multifaceted, subsuming behaviors, values, and attitudes that are dominant and unique to a particular group of people [10]. There are also multiple theories that cover distinct elements of these cultural constructs. News media has a strong relationship with many macro-level factors in society, ranging from the economy, governments, the public, and other organizational structures [5,11]. Similarly, the pandemic exerts a large collective impact on multiple facets of lives at a global level. As such, our investigation places emphasis on the cultural characteristics of processes at the group or national level, such as public norms, organizational behaviors, and corporate structures. This is opposed to theories that place greater weight on cultural values about coordinated social interactions, or shared values about goal-directed behaviors (e.g., Schwartz cultural values) [12,13,14]. Based on this backdrop, we utilized seminal work by Geert Hofstede [9,15] to understand how the diversity of COVID-19 news relates to a cooperative framework of cultural values.

Hofstede presents a framework comprising the following six cultural dimensions at a national level [9,15]: power distance, individualism vs. collectivism, uncertainty avoidance, masculinity vs. feminism, long-term orientation, and indulgence vs. restraint. As society falls back on its default cultural orientations during crises [16], this guides their responses to risks such as pandemics. Studies have found that some cultural values are associated with death rates [17], safe-distancing behaviors [18], and mental resilience [16]. Consequently, a society’s cultural values can impact the rates of cases, fatalities, and lockdowns. Naturally, the media would follow suit and pursue these changes, influencing the degree of diversity in the news about the pandemic. Simultaneously, culture is intrinsically embedded in the production of mass media, and is described as a reflection of a nation’s cultural orientation [11,19]. A recent study on a corpus of COVID-19 news articles concluded that power distance (PD), individualism, uncertainty avoidance (UA), and long-term orientation (LTO) predicted the volume of coverage for the pandemic, represented by the frequency of COVID-19 keywords [20]. However, whether cultural values would have the same impact for diversity in the way COVID-19 is presented in the media remains unknown.

Diversity in the media can be assessed by the linguistic variation in the way the pandemic is expressed [21]. Underlying frames and discourses are typically deduced by extracting topical analyses from a given text, which stem from a corpus using collocations; for instance, a recent study that assessed collocates of pandemic keywords revealed varied linguistic representations of COVID-19 in the news media [2]. These variations, therefore, gave rise to a novel interpretation of issues pertaining to the pandemic [2]. Another study uncovered topical fluctuations about the pandemic across twenty countries in an online news corpus [22]. Utilizing collocates of pandemic keywords, diversity peaked between March and May 2020, with five identified topics (i.e., testing, societal risk and vulnerability, mitigations and lockdowns, economy, deaths) [22]. Therefore, diversity in the mass media for the pandemic can be taken as the degree of linguistic variation related to key COVID-19 target words.

To our knowledge, this is a first study to test for the relationship between cultural values and the diversity of the way COVID-19 is represented in the media. The mass media commands a forceful influence on society’s perceptions; hence, our study potentially informs the position of culture in media theories and practices in relation to public policies. Previous findings have implicated the influence of PD, individualism, UA, and LTO on the volume of pandemic articles [20]. This paves the way to further explore the relevance of cultural values for the diversity in the way COVID-19 is depicted in the mass media. As there is little past empirical work on the intersection between culture and media diversity for the pandemic, we take an exploratory stance, broadly hypothesizing that cultural values would predict the amount of diversity in the way COVID-19 is presented in the media.

## 2. Materials and Methods

### 2.1. Corpus Data

Media data were taken from the news on the web corpus [23]. This is a large collection of online news, amassing a total of 7000 English articles. The corpus is updated monthly, with 200 million words from 300,000 articles. The articles are obtained from 20 countries, spanning the following six regions: America, Canada (North America); Australia, New Zealand (Oceania); Bangladesh, Hong Kong, India, Malaysia, Pakistan, Philippines, Singapore, Sri Lanka (Asia); Ghana, Kenya, Nigeria, South Africa, Tanzania (Africa); Ireland, United Kingdom (Europe); Jamaica (Caribbean). The corpus includes both local and international media news outlets, with a wide range of topics. For instance, global networks include *BBC*, *CNN*, and *CNBC*. Local news networks such as *Leicester Mercury*, *The Guardian*, and *Manchester Evening News* from the UK are also recorded. This dataset was created with funding from the National Science Foundation (NSF) and the National Endowment for the Humanities (NEH) to study contemporary language usage in countries where English is widely used.

### 2.2. Outcome Variable: Media Diversity

To track the diversity of pandemic-related media coverage, we identified 10 target words—coronavirus, COVID-19, COVID, nCoV, SARS-CoV-2, Wuhan virus, virus, disease, epidemic, pandemic—and compiled all sentences containing each of these target words from October 2019 to May 2020, culminating 1.5 billion words. While we acknowledge that terms such as ‘Wuhan virus’ have been politicized, they were included as we needed to capture early articles from December 2019 to February 2020. After pre-processing the dataset by excluding prepositions, conjunctions, and ‘stop’ words (e.g., and, the, that), we generated collocates (i.e., words that co-occurred most frequently with 10 target words) for each of the 20 countries, every week, between October 2019 and May 2020 (total of 25 weeks). These collocates had the following qualifying criteria: (a) Lexical proximity: collocate present within six words prior to or after the respective target word. Articles such as ‘the’ and ‘a’ were not included in the six-word lexical span. If the target noun was the first word of a sentence, the collocates from the prior sentence were excluded; (b) mutual information (*MI*) score estimates word association norms directly from the corpus. It is calculated via sentiment analysis, which shows the mutual information between collocates and target words. The higher the *MI* value, the closer the relationship between the collocate and target word. The *MI* value is calculated using the following formula:

$$MI=\frac{log\left[\frac{C*SizeCorpus}{A*B*Span}\right]}{log2}$$

‘*A*’ indicates the possibility of the target word *A* appearing, which is calculated by the frequency of the target word. ‘*B*’ indicates the possibility of the collocate *B* appearing, which is calculated by the frequency of word *B*. ‘*C*’ indicates the possibility of ‘*A*’ and ‘*B*’ appearing together, which is calculated by the frequency of collocate *B* appearing near the target word *A*. ‘*SizeCorpus*’ refers to the size of corpus or the number of words. Span is the span of words (e.g., if there are 6 words to the left and 6 words to the right of the target word, span = 12). This is an application of computational linguistics to study topic content and language shifts in many studies [10,23,24]. The rigorous process culminated 1,918,794 unique collocates—representing the diversity of media coverage. Specifically, the formula for media diversity rate was as follows: (Total unique collocates for all countries for the respective week)/(Total corpus size for all countries for the week) × 1,000,000.

### 2.3. Pandemic Variables

COVID-19 variables were included in our analyses to ascertain the relative importance of cultural values towards diversity in the media. For this, data were obtained from the Oxford COVID-19 government response tracker [24]. This includes data from 77 countries, with weekly updates of the following several indicators of restrictions: workplace, school, and public transportation closures; cancellation of public events; restriction on gatherings; stay-at-home notices; internal and international movement; public informational campaigns; testing policies; fiscal and monetary measures; investment in healthcare and vaccines; contact tracing. These indicators are combined and weighted (by country), as per the original methodology provided, forming a single governmental stringency index (GSI).

The definitions of the pandemic variables taken from the Oxford COVID-19 government response tracker are as follows: *COVID-19 velocity* is the rate of increase in new cases presented as a percentage. This was calculated from the number of new cases for the respective week in each country, divided by the total number of COVID-19 cases from the previous week, multiplied by 100. *COVID-19 prevalence rate* is the proportion of the population in a country infected with COVID-19. This was ascertained by taking the cumulative number of COVID-19 cases for the respective week, divided by the respective country’s population, multiplied by 100,000. *COVID-19 mortality risk* is the rate of new deaths represented as a percentage. This was calculated by dividing new deaths for the respective week in the respective country by the total deaths from the previous week, multiplied by 100. *Cumulative mortality risk* is the proportion of deaths in each country’s population. The number of deaths for the respective week is divided by the respective country’s population, multiplied by 100,000.

### 2.4. Cultural Values

Data for cultural values are publicly available, and obtained from a cross-national survey of Hofstede’s cultural dimensions using the values survey module (2013) [25,26]. The survey consists of 30 items; there are 4 questions for each of the 6 cultural dimensions (i.e., 6 × 4 = 24 content questions), plus 6 questions pertaining to demographic information. First, individual responses were calculated at a national level. Next, national scores were averaged and weighted, providing a profile of cultural values for each country (0 to 100). Responses were on a 5-point Likert scale, and dichotomous items and multiple-choice questions were combined and calculated on a percentage basis. National-level scores were weighted, and a score for each dimension for each country ranged from 0 to 100.

*Power distance* (PD) deals with the inequality of the distribution of power perceived by those from the lower stratum. Societies with high PD accept greater disparity of power than those with low PD. *Uncertainty avoidance* (UA) is the degree to which society tolerates ambiguity. Countries with high UA usually employ strict regulations and are generally uncomfortable with unstructured environments. *Individualism* vs. *collectivism* are opposites; the former stresses individual freedom and uniqueness, and the latter focuses on placing the interest of the group before themselves, emphasizing conformity and interdependence. The current data measure the degree of individualism for each country. *Masculinity* vs. *femininity* are opposites representing the distribution of gender roles in society, but these also extend to other characteristics. Masculine societies value competitiveness and material success. Feminine societies stress modesty and the importance of social welfare. The current data were assessed for the degree of masculinity for each country. Societies high on *long-term orientation* (LTO) foster future-oriented rewards, perseverance, thrift, and value adaptability. *Indulgence* vs. *restraint* was a later addition, and this is generally concerned with the level of free gratification of human desires. The current data measure the level of indulgence.

Hofstede demonstrated criterion validity by correlating different cultural dimensions with other indicators that were theoretically expected to be aligned with these values [9,25]. PD, for instance, found positive correlations with political domestic violence and extremism as well as income inequality in a country [9]. Individualism correlated with national wealth and the movement of people between social classes [9]. Masculinity found negative correlations with the amount governments from wealthy countries spent on developing third-world countries [9]. Long-term orientation received positive correlations with national savings [9]. In other reports, Hofstede found that Cronbach’s alphas were >0.7 for PD, individualism, masculinity, and UA across 40 countries [25]. Scores across these dimensions also demonstrate temporal stability over a 45-year period across different countries [27].

### 2.5. Analytic Strategy

Linear mixed regression was conducted to ascertain the predictive value of cultural values with week as the random effect. Multicollinearity was assessed with VIF tolerance scores with a conservative criterion of less than 5. Changes in the predictive values of pandemic variables and cultural value variables were assessed by progressively conducting three regression models. The first model included pandemic variables (Model 1), followed by the GSI (Model 2), and finally all cultural values were entered into the model (Model 3). All analyses were conducted on R.3.2.3 (Insightful, Seattle, WA, USA).

## 3. Results

### 3.1. Descriptive Statistics

**Figure 1:**
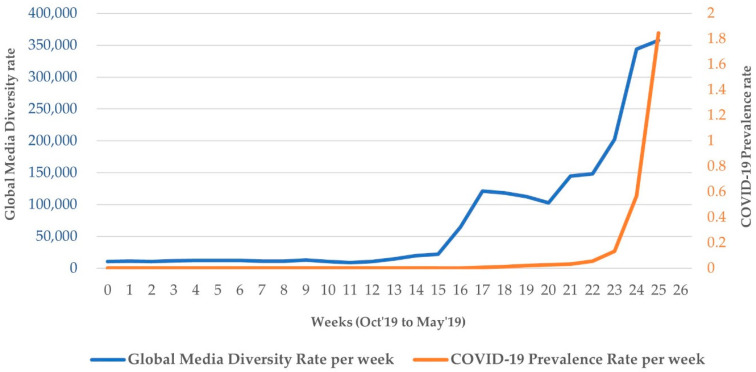
Global media diversity rate and COVID-19 prevalence rate over 25 weeks from a baseline period (October–December 2019) to during the pandemic (January–May 2020).

**Figure 2:**
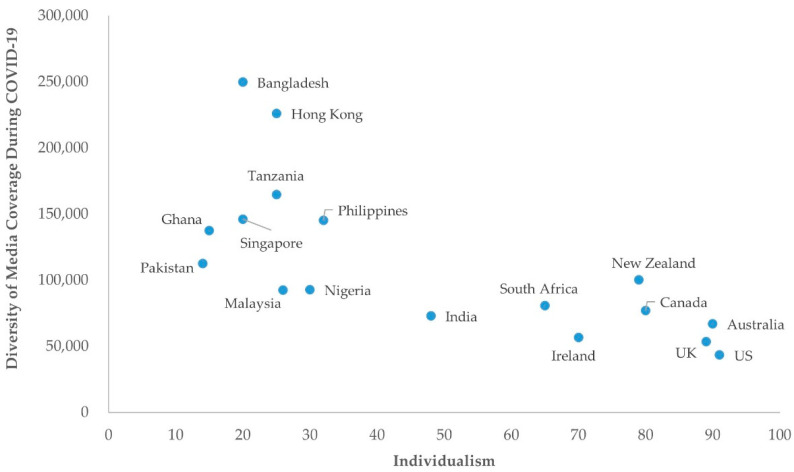
Scatterplot for diversity of media coverage during COVID-19 and individualism scores for 17 countries.

Global media diversity grew at a rate of 6.7 times over 25 weeks (see Figure 1). All the COVID-19 indicators increased as the pandemic deepened. Scatterplots revealed interesting relationships between cultural values and media diversity; specifically, Figure 2 shows an inverse relationship between individualism and the diversity of COVID-19 media coverage. Collectivist countries tend to have more diverse COVID-19 media coverage.

### 3.2. Mixed Effects Regression

Three regression models (Table 1) were implemented to ascertain the unique contribution of cultural values to the diversity of COVID-19 in the media. In the first model, only a cumulative number of cases were significant (B = −41,102.6, *p* < 0.01). Next, the GSI (B = 179.54, *p* < 0.01), number of cases (B = −39,514.7, *p* < 0.01), and rate of new cases (B = −18.96, *p* = 0.047) were significant in predicting the number of collocates. In the final model, a cumulative number of cases (B = −3529.9, *p* < 0.01), GSI (B = 156.50, *p* < 0.01) and individualism (B = −32.23, *p* = 0.012) significantly predicted the diversity of global media coverage on COVID-19, providing support for our hypothesis. Specifically, countries low in individualism (i.e., collectivist countries) evidenced more diverse COVID-19 media coverage, controlling for pandemic severity and government stringency.

Broadly, the results supported our cultural hypothesis, though only individualism evidenced a negative relationship with media diversity. We also found that government stringency (measured by Oxford’s GSI) and pandemic severity predicted media diversity. Specifically, as COVID-19 incidence increased, the diversity of media coverage decreased. This could reflect a convergence in topics, where the media focuses on specific issues that are most pertinent, resulting from the surge in COVID-19 cases [22]. This is one of the first known studies that investigates the relationship between cultural values and the diversity of COVID-19-related media coverage.

## 4. Discussion

Our findings demonstrate the application of Hofstede’s theory, positioning its relevance towards media diversity about the pandemic. Cultural dimensions provide a context in explaining relevant news frames across the world. Hofstede theorized that higher individuality ought to result in greater media coverage, citing that these societies value diverse opinions and the means to disseminate those perspectives [15]. This is, in part, due to greater press freedom that is associated with highly individualistic societies [9]. As a result, greater journalistic freedom from individualistic countries can result in greater diversity of news topics and content [28,29]. At the same time, the media has been described as a vessel that can induce change, especially in repressive societies [3]. This means that the value of power distance may have some implications for media diversity production [3]. Contrary to these claims, it seems that only the dimension of individualism within Hofstede’s framework is important towards media diversity. Perhaps other cultural values, such as PD, may be more salient towards other aspects of media production and dissemination.

According to our findings, the negative relationship between individualism and the diversity of COVID-19-related media coverage is counterintuitive. As mentioned, media from highly individualistic societies typically produce a greater range of opinions and topics for issues in society [9,28,29]. With greater journalistic expression, this ought to result in greater media diversity. Our results were contrary to these claims; perhaps these societies that provide a greater range of news topics may not necessarily produce diversity within the confines of the pandemic. Alternatively, it is possible that media outlets in less individualistic societies are inclined to harness conformist and collectivistic sentiments in society, and this could be reflected in the way they deal with the media coverage of the pandemic [7]. Low individualism is usually indicated by a strong sense of conformity and placing group interests above individual goals. As a result, these outlets may push for reports to cover a wider range of issues that are pertinent to the pandemic to draw collective attention to the impact of the pandemic. Alternatively, the results herein could simply reflect the nature of media involvement during a crisis. During such events, news media is more likely to align with governments, perhaps presenting the public with a common narrative [30]. Perhaps cultural values may have a mediating role in the relationship between authorities and the production of news. Further studies may consider conducting qualitative analyses regarding the intervening nature of individualism, regarding media diversity and the characteristics of the pandemic.

This paper contributes to the literature on the role of culture in news and media research in two ways. First, individualism is important for the degree of diversity in the news about the pandemic, but a wider set of cultural values (i.e., PD, individualism, LTO, UA) may play a larger role in the quantity of coverage for COVID-19 [20]. This might be informative for the agenda theory to distinguish between the amount of coverage from the content of news information and its differential effects on public perceptions [5]. Second, this study lays the groundwork to inform media outlets and decision makers about the importance of cultural values in the way COVID-19 is presented to the public. Based on these findings, a society’s individualistic orientation can be accounted for during the development of policies for communicative practices during the pandemic; for instance, harnessing collective sentiments through media coverage can develop societal resilience during such crises [7]. The results here suggest that highly individualistic cultures are related to a greater range of perspectives on the pandemic. Decision makers might need to consider how the individualistic nature of society and the range of media perspectives impact public health behaviors.

### Limitations

Our study, however, is not without limitations. As our data are limited to English language news reports, they may not generalize to non-English-speaking media outlets. Conceptually, we were not able to include all national values, but this was done to maximize the number of countries and cultural dimensions. For this, we suggest that future researchers examine the role of cultural values [30] over a wider range of countries, national values, methodologies and across different language news sources. Furthermore, diversity can exist within and between news outlets, and differs with the level of media sources (i.e., type of medium, news bundle programs, individual news units, etc.). As such, future research could explore how national values change as a function of different types of media sources regarding the pandemic; for example, national values may explain the variance in media diversity between countries or news sources, but this may change for diversity within a single news outlet or network.

## 5. Conclusions

Our study explored the relationship between media diversity about the pandemic and its underlying cultural values. We demonstrated how Hofstede’s framework can be applied to media diversity, specifically, higher collectivism was related to higher media diversity. This could be attributed to the collective nature of society and news outlets that are motivated to present a diverse array of topics given COVID-19’s impact on every segment of society. Of broader significance, this is one of the first known studies to operationalize media diversity and link it to cultural values. Our paper, therefore, contributes to communication theories, highlighting how different cultural values affect different aspects of media production (i.e., content vs. volume, etc.). We also hope to have laid the groundwork for designing targeted public health communications that are culturally nuanced. Based on this backdrop, we proposed that policy makers consider society’s cultural values to influence public behaviors and perceptions of the pandemic.

## Figures and Tables

**Table 1:** Presents three mixed effect regression models with prevalence of collocates as the outcome, with fatalities, cases, and GSI as covariates.

		B	SE	*p*	Sig	R^2^
Model 1						0.71
	COVID-19 Velocity ^a^	−8.79	10.21	0.389		
	COVID-19 Prevalence rate ^b^	−41,102.61	9197.85	<0.01	**	
	COVID-19 Mortality rate ^c^	−2.32	11.10	0.835		
	Cumulative mortality risk ^d^	−368,549.13	336,619.03	0.274		
Model 2						0.76
	COVID-19 Velocity ^a^	−18.96	9.56	0.047		
	COVID-19 Prevalence rate ^b^	−39,514.69	8484.72	<0.01	**	
	COVID-19 Mortality rate ^c^	−21.65	10.41	0.038		
	Cumulative mortality risk ^d^	−33,6557.03	309,757.13	0.277		
	Government Stringency Index ^e^	179.55	18.38	<0.01	**	
Model 3						0.78
	COVID-19 Velocity ^a^	−8.17	9.29	0.379		
	COVID-19 Prevalence rate ^b^	−35,269.90	8227.38	<0.01	**	
	COVID-19 Mortality rate ^c^	−13.70	10.00	0.171		
	Cumulative mortality risk ^d^	−273,496.64	296,283.48	0.356		
	Government Stringency Index ^e^	156.51	19.08	<0.01	**	
	Power Distance	−3.32	13.22	0.802		
	Individualism	−32.24	12.87	0.012	*	
	Masculinity	−42.88	27.49	0.119		
	Uncertainty avoidance	11.13	16.15	0.491		
	Long Term Orientation	16.31	13.52	0.228		

^a^ Number of new COVID-19 cases for the respective week per country/total COVID-19 cases in the previous week × 100. Velocity is the rate of increase in new cases; represented as a percentage. ^b^ Cumulative number of COVID-19 cases for the respective week/respective country population × 100,000. ^c^ Rate of increase in new COVID-19 deaths. New COVID-19 deaths for the respective week in the respective country/total COVID-19 deaths in the previous week × 100; represented as a percentage. ^d^ Cumulative COVID-19 mortality for the respective week/respective country population × 100,000. ^e^ Extracted from the Oxford COVID-19 government response tracker. Variables are recorded weekly per country; they include workplace and school closures, closing of public transport, cancellation of public events, restriction on gatherings, stay-at-home restrictions, constrictions of internal and international movement, public informational campaigns, testing policies, fiscal and monetary measures, investment in healthcare and vaccines, and contact tracing. These indicators are weighted and combined into the government stringency index (GSI), ranging from 0 (least stringent) to 100 (most stringent). * *p* < 0.05, ** *p* < 0.01.

## Data Availability

Data are publicly available online at https://www.english-corpora.org (accessed on 1 January 2015).
